# The paradox of aging and health-related quality of life in Asian Chinese: results from the Healthy Aging Longitudinal Study in Taiwan

**DOI:** 10.1186/s12877-020-1446-y

**Published:** 2020-03-05

**Authors:** Han-Yun Tseng, Corinna Löckenhoff, Chun-Yi Lee, Shu-Han Yu, I-Chien Wu, Hsing-Yi Chang, Yen-Feng Chiu, Chao Agnes Hsiung

**Affiliations:** 1 Institute of Population Health Sciences, National Health Research Institutes, 35 Keyan Rd., Zhunan, Miaoli County 35053 Taiwan; 2 Department of Human Development, Cornell University, G60B Martha Van Rensselaer Hall, 116 Reservoir Ave, Ithaca, NY 14853 USA; 3 Aesthetic-Mind Clinic, 334 Dasheng St, Nantun District 408, Taichung City, Taiwan

**Keywords:** Mental health score, Physical health score, Objective physical health, subjective physical health

## Abstract

**Background:**

Declines in health, physical, cognitive, and mental function with age suggest a lower level of health-related quality of life (HRQoL) in late life; however, previous studies found that the associations were weak and varied, depending on the study designs and cohort characteristics.

**Methods:**

The present study examined the paradox of aging in an East Asian context by regressing the age patterns of objective health indicators (physical, cognitive, and mental function), and subjective HRQoL (12-item Short Form, SF-12), on the independent and interactive effects of age and physical function in a cohort study of 5022 community-dwelling adults aged 55 and older in Taiwan.

**Results:**

Age patterns differed across measures. The SF-12 mental health score (MCS) showed a slight positive association with age and this effect remained stable after controlling for various age-related covariates. The SF-12 physical health score (PCS), in turn, was negatively associated with age. Age differences in PCS were fully explained by age decrements in objective physical health. However, consistent with the so-called paradox of aging, the association between objective and subjective physical health weakened with age.

**Conclusion:**

These findings add to prior evidence indicating that — in spite of objective health decrements — subjective HRQoL is maintained in later life among Asian Chinese. Also, these paradoxical patterns appear to vary for mental and physical components of HRQoL, and future research is needed to explore the underlying mechanism.

**Trial registration:**

Healthy Aging Longitudinal Study in Taiwan (HALST) is retrospectively registered at ClinicalTrials.gov on January 24, 2016 with trial registration number NCT02677831.

## Background

Self-reported quality of life usually follows a U-shaped or curvilinear relationship over the course of the life cycle [13, 17] with the lowest points reported between the person’s mid-30s and early 50s in the United States, Canada, Britain, most West European countries, Australia, and New Zealand [4, 10, 11, 43]. The results have been found to be fairly robust for men and women [4, 22], and are seen in both raw and covariate-adjusted data [17]. One possible explanation for the lower quality of life in midlife is that deteriorating health, accumulated life stressors, and competing demands from work and family can be difficult and detrimental to well-being [7, 40].

However, what interested gerontological researchers, and was initially considered a mystery, was the robust upturn of subjective well-being found later in life even though health-related resources (e.g., health and function) are progressively lost. Researchers using longitudinal data of 22 waves from the Veteran’s Affairs Normative Aging Study found the highest well-being scores were reached in late life [34]. Similarly, in a cross-sectional analysis, when potential covariates (e.g., sex, education, income, driving ability, activities of daily living (ADL), social relationship, living status, and depression) were adjusted, researchers in the English Longitudinal Study of Ageing (ELSA) observed that the quality of life increased from age 50, peaked at age 68, and, by age 86, gradually declined to the same level as at the age of 50 [35]. Such consistent reports of stable or increased levels of subjective well-being among older adults also suggest that the correspondence between objective health and subjective well-being weakens with age [24]. That subjective well-being is maintained or even improved later in life despite so many well-documented losses that occur with aging has been labeled the “paradox of aging” [33].

Subsequent research has begun to unravel the increasing decoupling of subjective and objective health with age — the core of the paradox of aging. Life-course perspectives [3] explain this phenomenon by arguing that although losses in objective health and socioeconomic status are apparent with aging, the effects of considerable reserves and continued gains in psychosocial functioning on quality of life are also noticeable [41]. The latter were found to compensate for losses in health even until the very last phase of life [27]. Prior studies have also documented a relevant range of covariates that serve as challenges and resources in later life. For example, more favorable chair stand scores and walk times were associated with higher HRQoL [18]. Psychosocial factors, such as depression, have been consistently found to have a substantial influence on quality of life in older adults beyond physical function and health status [26]. Similarly, social relationships not only promote subjective well-being when one gets older [2, 5] but also explain over 20% of the variance in life satisfaction in old age [14]. Meanwhile, it is important to acknowledge that age-related shifts in risk and resilience factors may not function consistently across different demographic and social groups. Individual as well as cultural variance in predictors of subjective well-being requires further consideration [36].

Compared to the wealth of literature on age trajectories and the aging paradox of subjective well-being in the West, fewer studies in Asian populations are available, and it is not clear to what extent such patterns are universal [42]. Chen [9], using the Survey of Health and Living Status of the Elderly in Taiwan in 1989 and 1993, revealed that, after controlling for age-related correlates, higher subjective well-being was found among older versus middle-aged birth cohorts. Likewise, researchers in China found the familiar U-shaped relationship between age and subjective well-being in a Chinese sample aged 15–102 years [44]. To our knowledge, none of these studies in East Asian populations have explicitly tapped into the issue of the paradox of aging. It is also unknown if the cross-sectional age differences found in Chen’s study 20 years ago would apply to adults currently aging in Taiwan, where significant social, economic, and demographic changes have been observed in the new millennium [30].

### The present study

The present study explored the paradox of aging in health-related quality of life among a large, recent, and nationally representative Taiwanese sample by investigating cross-sectional age patterns in HRQoL and examining age differences in the association between objective physical functioning and HRQoL.

Several methodological improvements are worth mentioning. First, rather than relying on single-item assessments of HRQoL, we used the well-established SF-12 scale, which has been shown to be valid across cultures [28] and captures the multidimensional nature of HRQoL by assessing both physical domains (physical component score, PCS) and mental domains (mental component score, MCS) of quality of life [19, 31]. Moreover, rather than relying on self-reported measures of health status, which may be subject to social comparison and appraisal processes, we utilized objective, performance-based measures of physical function, along with well-established, standardized questionnaires for other health covariates. Finally, we systematically compared the age patterns of HRQoL (PCS and MCS, respectively) before and after controlling for a range of relevant demographic, socioeconomic status (SES), and health covariates in order to gain a full picture of actual and pure age effect [22].

We depicted the cross-sectional age patterns of objective health indicators (physical, cognitive, and mental function) and HRQoL (PCS and MCS, respectively). According to the paradox of aging, we expected to see divergent age patterns, including decrements in PCS but stable, if not improved, MCS. It is of particular interest in the current study to see if age would remain a significant predictor for HRQoL when age-related changes were considered. Further, we examined the association between an *objective* health indicator (physical function) and *subjective* HRQoL and how it varied as a result of age. We hypothesized that, with advancing age, the predictive value of physical function for HRQoL would be minimized. In other words, the effects of objective health on subjective HRQoL (PCS and MCS) are moderated by age.

## Methods

### Data and sample

**Fig. 1:**
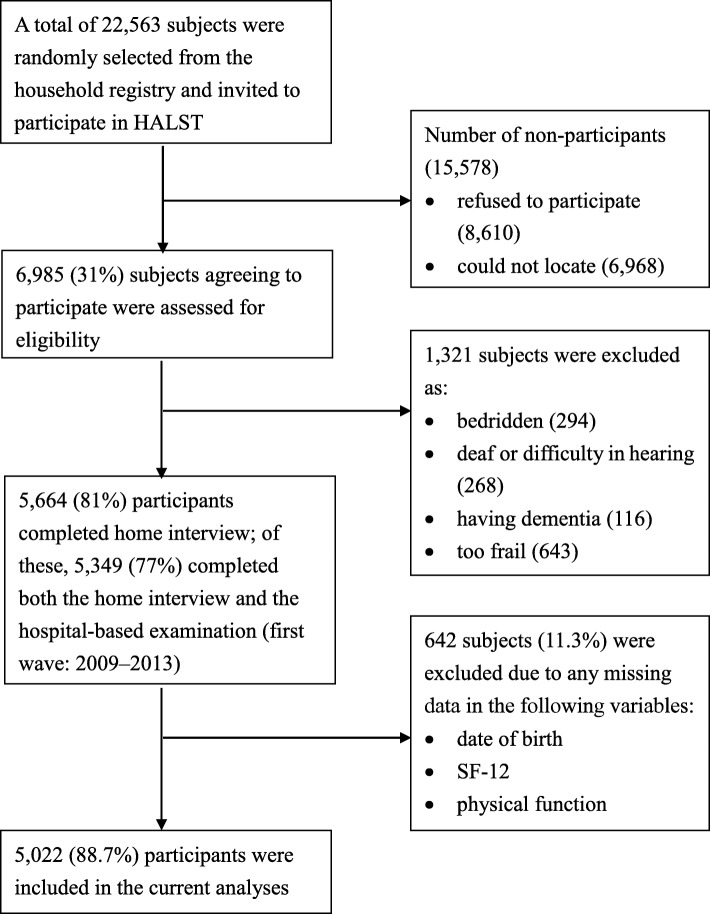
A flowchart of the data-collection process and attrition for the HALST study (baseline: 2009–2013), and the data selection for the current study (*n* = 5022)

The data was obtained from the baseline sample of the Healthy Aging Longitudinal Study (HALST; for details, see Hsu et al.) [23]. The baseline sample was collected in 2009–2013 from community-dwelling older adults aged 55 and above across Taiwan (*n* = 5664; age range = 55.1–104.2 years, mean = 69.6 years, SD = 8.3; 52.8% female). The sampling distributions of gender and education attainments in the HALST study represented the socio-demographic distributions of Taiwanese population in urban and rural areas. Individuals who had any of the following four conditions were excluded from the study: (1) bedridden, (2) deaf or difficulty hearing, (3) demented, and (4) too frail. Figure 1 presents a flowchart of the data collection process for the HALST and sample selection for the current study.

### Measures

#### Outcome variables

##### Health-related quality of life

Health-related quality of life was assessed with the SF-12v2 [46]. Compared to the longer version (i.e., 36-item Short Form (SF-36)), the SF-12v2 is better suited for older adults because it is less time consuming and excludes questions about work [32]. Previous studies have demonstrated the sound psychometric properties [45] and cross-cultural compatibility of the SF-12 [28], and it is considered suitable for application to Asian populations. The traditional Chinese version of the SF-12v2 was obtained through licensing from QualityMetric (http://www.qualitymetric.com/). The SF-12v2 includes two questions concerning physical function, two questions on role limitations due to physical health problems, one question on bodily pain, one question on general health perceptions, one question on vitality, one questions on role limitation due to emotional problems, and two questions on psychological distress and well-being. Scoring followed the standard SF-12 algorithms in the user’s manual for the SF-12v2 [46], yielding two aggregated summary measures: the physical component summary score (PCS) and mental component summary score (MCS).

#### Predictive and controlled variables

Several age-related covariates for HRQoL were selected for the present study based on the results of prior research in China, Hong Kong, and Taiwan [28, 29, 44], including health status, physical function, social support, and a range of socio-demographic variables.

##### Physical function

Performance-based mobility was measured by the Short Physical Performance Battery (SPPB) [20, 21]. The SPPB contains three components: standing balance, a timed 4-m walk, and chair stands. The standing balance activity required participants to maintain side-by-side, semi-tandem, and tandem stances for 10 s. The 4-m walk took place twice, and the faster time of the two attempts was used. The chair stands activity asked participants to rise from a chair five times with arms across their chest. Times for completing the 4-m walk and chair stands were categorized into quintiles, ranging from 0 to 4. Individuals unable to complete the task received a score of 0. The sum of the three components comprised the final SPPB summary score, with a possible range from 0 to 12 — the higher the score, the better the physical function.

##### Cognitive function

The Mini-Mental State Examination (MMSE) [16] was used to screen and assess cognitive function, including memory, attention, and language. The standard MMSE form is currently used worldwide with minor modifications to adapt to differences in language and cultural conceptualization of the questions. The MMSE has been used extensively in clinical population-based research to measure cognitive function, estimate the severity and progression of cognitive impairment, track the course of cognitive change over time, and screen for dementia [12]. MMSE scores range from 0 to 30, with higher scores indicating better cognitive function.

##### Mental function

The self-reported Center for Epidemiologic Studies Depression Scale (CES-D) [37] was used to measure depressive symptoms severity in the general population. The CES-D contains 20 items regarding symptoms that occurred in a week prior to the interview. Each response could range from 0 to 3, indicating the frequency of the described symptoms. The final score ranges from 0 (best possible) to 60 (worst), and the cut-off point recommended for depression is 16.

##### Socio-demographic variables

A range of socio-demographic factors, which have been shown to be associated with HRQoL, were selected based on the literature. These included levels of education (0 = illiterate, 1 = elementary school, 2 = middle school or higher), residence (0 = rural, 1 = urban), employment (0 = unemployed, 1 = employed), living arrangement (0 = living alone, 1 = cohabitant), partnership status (0 = single, widowed, divorced, or separated, 1 = married or partnered), and gender (0 = male, 1 = female).

### Analytic strategy

The first analysis was descriptive, and categorized participants by demographics, socioeconomic, objective health indicators, and HRQoL. The Kruskal–Wallis or chi-square test was used to examine the difference in continuous or categorical variables of interest between the young old and the older old (≥65 years old). The second analysis used multiple linear regression models predicting PCS and MCS. Models included: (1) age (tested by linear, quadratic, and cubic fashion for best fit); (2) as previous, plus demographic, SES, and covariates, including gender, education level, partnership, residency, living arrangement, MMSE, and CES-D; (3) as previous, plus objective health indicators, including handgrip strength and SPPB; (4) as previous, plus interaction terms of age and objective health indicators. All analyses described in the following section were performed using SAS version 9.4 statistical software (SAS Institute Inc., Cary, NC, USA). LOESS (LOcally regrESSion) smoothing method was used to model the association between age and PCS or MCS. The fitted curves were plotted using R computing package.

## Results

Table 1 presents descriptive statistics for the variables under consideration in the present study. The mean scores for PCS and MCS in our sample are 46.8 ± 0.1 and 59.3 ± 0.1, respectively. The average age is 69.6 ± 0.1 years (ranging from 55.1 to 104.2 years), and 52.8% are female. The vast majority of participants (85.9%) had at least an elementary school education level when leaving school. The mean MMSE score is 26.2 ± 0.05, which falls within the normal cognitive function. A total of 5.6% reported depressive symptoms in the past 2 weeks (CES-D > 16). The mean handgrip strength is 29.0 ± 0.1 (kg), which falls within the range of normal to strong upper limb strength. The average SPPB score is 10.4 ± 0.03 out of 12.

**Table 1:** Descriptive statistics. Mean (SE) or proportions (%)

Characteristics	All	Young old (< 65 y)	Old old (≥65 y)
N (%)	5664 (100.0)	1686 (29.8)	3978 (70.2)
Age (mean ± SE)**	69.6 ± 0.1	59.8 ± 0.06	73.8 ± 0.09
Sociodemographic characteristics			
Female (%)	2988 (52.8)	886 (52.6)	2102 (52.8)
Education level**			
Illiterate	799 (14.1)	49 (2.9)	750 (18.9)
Elementary school	2322 (41.0)	570 (33.8)	1752 (44.1)
Middle school or higher	2539 (44.9)	1065 (63.2)	1474 (37.1)
Employed (%)**	1433 (25.3)	783 (46.4)	650 (16.3)
Partnered (%)**	4159 (73.4)	1438 (85.3)	2721 (68.4)
Reside in urban area (%)	2911 (51.4)	891 (52.8)	2020 (50.8)
Cohabitant (%)**	5155 (91.0)	1579 (93.7)	3576 (89.9)
Health-related variables			
Handgrip strength (mean ± SE)**	29.0 ± 0.1	33.1 ± 0.2	27.3 ± 0.2
SPPB (mean ± SE)**	10.4 ± 0.03	11.4 ± 0.03	10.0 ± 0.05
MMSE (mean ± SE)**	26.2 ± 0.05	28.0 ± 0.06	25.4 ± 0.06
Depression (CES-D > 16; %)	318 (5.6)	98 (5.8)	220 (5.5)
HRQoL			
PCS (mean ± SE)**	46.8 ± 0.1	49.3 ± 0.2	45.7 ± 0.2
MCS (mean ± SE)**	59.3 ± 0.1	58.7 ± 0.2	59.6 ± 0.1

Kruskal–Wallis test or ${\chi}_2^2$ test was conducted to detect the differences between two groups (Young old, and Old old)

* *p*-value< 0.05, ** *p*-value< 0.001

^*a*^ Urban: *City: Taipei, Changhua, Kaohsiung, Hualien *Rural: Miaoli, Yangmei, Shoufeng, Chiayi

**Fig. 2:**
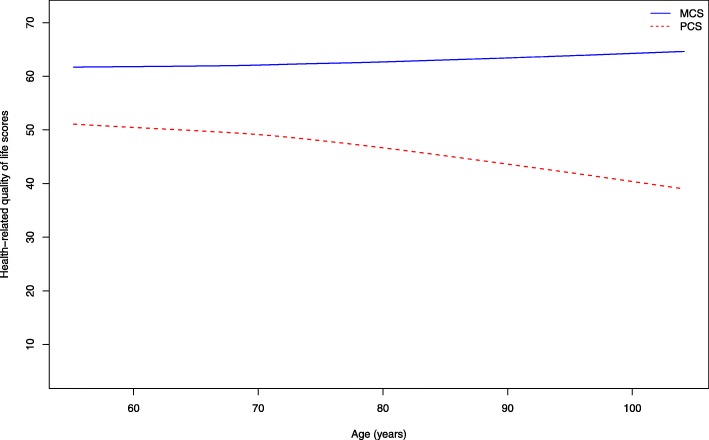
LOESS fitted MCS and PCS curves over age

We analyzed the zero-order correlations among variables of interest in our model first. As seen in Table 2, age was negatively associated with handgrip strength (*r* = −.33, *p* < .001), SPPB (*r* = −.42, *p* < .001), and PCS (*r* = −.26, *p* < .001), but slightly positively associated with MCS (*r* = .063, *p* < .001). Thus, older age was accompanied by more constraints in physical and cognitive function, and lower HRQoL in the physical domain (PCS), whereas HRQoL in the psychosocial domain (MCS) was preserved, if not slightly improved, with age. The divergent cross-sectional age trajectories of PCS and MCS before controlling for covariates are visualized in Fig. 2.

**Table 2:** Intercorrelations among age, health-related variables, and HRQoL (*n* = 5022)

Variables	Age	MMSE	CES-D	Handgrip strength	SPPB	PCS	MCS
Age	–	−0.40**	0.046*	−0.33**	− 0.42**	− 0.26**	0.063**
MMSE	−0.40**	–	− 0.18**	0.39**	0.43**	0.26**	0.049**
CES-D	0.046*	−0.18**	–	− 0.18**	−0.24**	− 0.25**	−0.67**
Handgrip strength	−0.33**	0.39**	−0.18**	–	0.40**	0.32**	0.066**
SPPB	−0.42**	0.43**	−0.24**	0.40**	–	0.55**	0.082**
PCS	−0.26**	0.26**	−0.25**	0.32**	0.55**	–	−0.048**
MCS	0.063**	0.049**	−0.67**	0.066**	0.082**	−0.048**	–

Note: * *p*-value< 0.05, ** *p*-value< 0.001

Two further findings in Table 2 deserve mention. First, the zero-order correlation between PCS and MCS is small and negative (r = −.048, *p* < .001), corresponding to the literature suggesting independence of subjective well-being in different domains [15]. Second, while both PCS and MCS were significantly correlated with all objective health indicators, the coefficients of associations with objective health indicators for MCS were relatively small compared to that for PCS.

Multiple regression analysis was used to further examine the age patterns of PCS and MCS and their associations with the different sets of predictors. Table 3 shows the results for PCS. Age was negatively associated with PCS (unstandardized coefficient (B) = − 0.28, SE_B = 0.015, *p* < .001), even after accounting for sociodemographic, mental, and cognitive covariates (B = − 0.22, SE_B = 0.017, *p* < .001). In Model 3, which added objective health indicators, both handgrip strength and SPPB were positively associated with PCS (B = 0.11 & 1.63, SE_B = 0.018 & 0.050, ps < .001), whereas age effects were reduced to non-significance, suggesting a mediation relationship. Adding physical function accounted for an additional 17% of the variance in PCS. The final model, including the significant interaction between age and physical health status, suggested that the strength of the predictive association of objective health on PCS became weaker with advancing age.

**Table 3:** Regressing PCS, showing unstandardized regression coefficients

	PCS			
	model 1	model 2	model 3	model 4
Age	−0.28 (0.015)**	−0.22 (0.017)**	− 0.025 (0.017)	0.055 (0.059)
Covariates				
Female (ref. male)		−1.73 (0.26)**	0.34 (0.33)	0.33 (0.33)
Elementary school (ref. illiterate)		−0.47 (0.41)	−0.45 (0.36)	− 0.48 (0.36)
Middle school or higher (ref. illiterate)		0.30 (0.47)	0.27 (0.42)	0.20 (0.42)
Employed (ref. unemployed)		1.45 (0.29)**	0.86 (0.26)**	0.87 (0.26)**
Partnered (ref. unpartnered)		−0.39 (0.31)	− 0.64 (0.27)*	−0.67 (0.28)*
Living in urban areas (ref. rural)		1.38 (0.24)**	1.15 (0.21)**	1.12 (0.21)**
Cohabitant (ref. alone)		−1.34 (0.44)*	−0.92 (0.39)*	−0.91 (0.39)*
MMSE		0.22 (0.043)**	−0.080 (0.039)*	−0.088 (0.039)*
CES-D		−0.31 (0.019)**	−0.18 (0.018)**	− 0.17 (0.018)**
Objective health indicators				
Handgrip strength			0.11 (0.018)**	−0.18 (0.098)
SPPB			1.63 (0.050)**	3.07 (0.42)**
Interaction terms				
Age*handgrip				0.0043 (0.0014)*
Age*SPPB				−0.020 (0.0056)**

Note: *N* = 5022. *p-value< 0.05, ** *p*-value< 0.001. Model 1: age (unadjusted); model 2: same as model 1 plus demographic, SES, MMSE, and CES-D variables; model 3: same as model 2 plus objective health indicators; model 4: same as model 3 plus interactions between age and objective health indicators. Preliminary analyses did not find any evidence for quadratic or cubic age terms (*p*s > .05); therefore, subsequent analyses included only a linear age term

Table 4 shows the results for MCS. Age was positively associated with MCS (B = 0.062, SE_B = 0.014, *p* < .001). This linear age effect became slightly stronger after accounting for covariates (B = 0.075, SE_B = 0.012, *p* < .001). In Model 3, the linear effect of age remained significant once physical function was accounted for (B = 0.063, SE_B = 0.014, *p* < .001). These results suggested that the impact of physical function on MCS did not differ among younger or older age groups. As seen in Model 3, a higher MCS score was also found among those with older age (B = 0.063, SE_B = 0.014, *p* < .001), illiteracy (as compared to middle or higher degree levels (B = − 1.02, SE_B = 0.35, *p* < .05), fewer depressive symptoms (B = − 0.93, SE_B = 0.015, *p* < .001), and worse lower limb function (B = − 0.15, SE_B = 0.041, *p* < .001).

**Table 4:** Regressing MCS, showing unstandardized regression coefficients

	MCS			
	model 1	model 2	model 3	model 4
Age	0.062 (0.014)**	0.075 (0.012)**	0.063 (0.014)**	0.050 (0.049)
Covariates				
Female (ref. male)		0.14 (0.19)	0.13 (0.27)	0.13 (0.27)
Elementary school (ref. illiterate)		− 0.55 (0.30)	−0.54 (0.30)	− 0.54 (0.30)
Middle school or higher (ref. illiterate)		−1.03 (0.35)*	−1.02 (0.35)*	−1.01 (0.35)*
Employed (ref. unemployed)		0.086 (0.21)	0.13 (0.21)	0.13 (0.21)
Partnered (ref. unpartnered)		−0.068 (0.23)	−0.042 (0.23)	− 0.038 (0.23)
Living in urban areas (ref. rural)		0.26 (0.18)	0.28 (0.18)	0.29 (0.18)
Cohabitant (ref. alone)		−0.24 (0.32)	−0.27 (0.32)	− 0.27 (0.32)
MMSE		−0.034 (0.032)	−0.011 (0.032)	− 0.0094 (0.032)
CES-D		−0.92 (0.014)**	−0.93 (0.015)**	− 0.93 (0.015)**
Objective health indicators				
Handgrip strength			0.0021 (0.014)	0.044 (0.081)
SPPB			−0.15 (0.041)**	−0.36 (0.35)
Interaction terms				
Age*handgrip				−0.00062 (0.0012)
Age*SPPB				0.0029 (0.0046)

Note: *N* = 5022. *p-value< 0.05, ** *p*-value< 0.001. Model 1: age (unadjusted); model 2: same as model 1 plus demographic, SES, MMSE, and CES-D variables; model 3: same as model 2 plus objective health indicators; model 4: same as model 3 plus interactions between age and objective health indicators. Preliminary analyses did not find any evidence for quadratic or cubic age terms (*p*s > .05); therefore, subsequent analyses included only a linear age term

### Results not shown in the text

Preliminary analyses did not find any evidence for quadratic or cubic age terms (ps > .05). Also, there was no significant gender difference found in the regression analysis. Therefore, subsequent analyses included only a linear age term and did not present a gender-split view of results. In addition, we excluded those with missing data in the main variables (*n* = 644, 11.3%). To get a closer look at the potential effect of missing not at random on the present analyses, compared to the included/final sample, those excluded from the analyses were younger, less likely to be female, more likely to be employed, residing in urban areas, and have higher MMSE scores. There were no significant differences in the distributions of the primary outcome variables (PCS and MCS) and any objective health indicators between the included and excluded samples.

## Discussion

The present study studied the aging paradox in quality of life by examining cross-sectional age patterns in HRQoL, as well as the role age plays in the associations between physical health and HRQoL (PCS and MCS, respectively).

### Divergent age patterns of HRQoL in old age

The present study described the cross-sectional age patterns of HRQoL. Consistent with our hypothesis, zero-order correlations showed divergent age trajectories for PCS and MCS components of HRQoL. For our sample, older age was accompanied by more constraints in the physical domains (PCS), whereas HRQoL in the psychosocial domain (MCS) was preserved, if not slightly improved, with age.

When the age-related HRQoL covariates were controlled, not surprisingly we found no association between lower PCS and advancing age. The results, particularly with PCS, were in agreement with prior concerns that the paradox of well-being revealed by adjusted data for sociodemographic and health status could lead to a false and over-optimistic view regarding preserved quality of life in late life [22].

The positive association between advanced age and MCS, on the other hand, was in line with theoretical perspectives emphasizing motivational and self-regulatory mechanisms that allow older adults to maintain psychosocial well-being in the face of health-related losses.

As our results revealed a small and negative correlation between PCS and MCS, it seems reasonable to assume that cross-sectional age patterns may vary by domains of subjective well-being [19, 31]. Our results also confirmed Kunzmann et al.’s [27] suggestion that more pronounced age difference in subjective well-being may be detected in domain- and time-specific assessments as opposed to context- and time-neutral questions.

Perhaps one of the most surprising results was that even with raw data (i.e., not adjusted for other background and health variables), we saw a small but significant upturn of MCS in our sample. In previous studies [9, 35, 44], the upturn of well-being and life satisfaction with age could be seen only after controlling for a series of SES- and health-related covariates. The relationship shown with the raw data deserves attention because it represents the actual age effect on HRQoL, considering all possible variations in every aspect of life as one ages.

Echoing prior research, it may not be realistic to expect a disease-free older age, considering the consistent cross-sectional associations found between age and constraints in physical function [25, 38]. Instead, the robust findings of relatively preserved psychosocial aspects of HRQoL reiterate the need for future research to examine the specific mechanisms that protect older adults from the negative impact of objective health decrements.

### The well-being paradox: associations among age, physical health, and HRQoL

The second objective of this study was to examine the association between *objective* health and *subjective* health-related QoL, and observe variations in the associations according to age. Consistent with our hypothesis, after controlling for covariates, the association between age and PCS was mediated by objective health status. The mediation pathways for age, objective health, and PCS suggested that physical health status, rather than age, is the best predictor for the inter- and intra-personal differences in PCS. Interestingly, however, the predictive value of objective health status for subjective QoL became weaker with advancing age in this Taiwanese older adults sample. This seemingly counterintuitive result corresponds to the paradox of aging phenomenon found in Western and highly industrialized countries.

In contrast to the analyses for PCS, the associations between MCS and objective health did not vary by age, and controlling for objective health did not further strengthen the slightly positive association between MCS and age. As noted in the introduction, variables beyond chronological age — including self-regulation, perceived control, social comparison, and social integration — may potentially affect the association between objective health and MCS, and should be explored in future research.

### Limitations

Our results need to be interpreted within the context of the study limitations. First, the cross-sectional nature of the study did not allow us to investigate longitudinal shifts in the associations among age and subjective HRQoL. Second, we were unable to determine if the observed age pattern was a result of cohort effects or a genuine representation of the aging effect. A longitudinal study would provide more insights into the relative role of age and into age-related differences in the effects of functional health on HRQoL, as well as cohort effects in HRQoL. Third, self-reported HRQoL may be subject to various kinds of bias, such as social desirability, that we were unable to evaluate with our dataset. We also acknowledged that self-reported health care and other disease-related data are important variables for HRQoL; however, they were not quite consistent with the insurance records. Thus, we included objective health-related variables as covariates. Fourth, it is worth noting that by focusing on community-dwelling adults a relatively healthier portion of the aging population in Taiwan have been selected. The results may not be generalizable to frailer older adults who are more likely to be institutionalized. Finally, the study was part of a larger cohort study [23], and the research question can be analyzed only within the boundaries of the original study.

## Conclusions

Notwithstanding these limitations, to the best of our knowledge, this is the first study with a representative cohort sample that used a well-validated measure of HRQoL to assess age differences in subjective well-being and to examine age-related shifts in the association between objective health and HRQoL, thus obtaining evidence for the paradox of aging in East Asia. Although our results may not be directly comparable to those of other well-established studies in the West (e.g., the ELSA, the Berlin Aging Study, the Veterans Affairs Normative Aging Study, the World Gallup Poll, the US General Social Survey, the British Household panel, Eurobarometer, and the National Health and Nutrition Examination Surveys), considering the differences in sample characteristics (age groups and health status), and measures (assessing happiness, positive or negative effect, and life satisfaction), our results showed similar conclusions that are complementary to these studies: The third age can be a period with high subjective quality of life.

Furthermore, these results are consistent with the aforementioned theoretical frameworks, suggesting mechanisms that allow for the preservation of HRQoL, especially MCS, in spite of objective health detriments among older adults. Future studies should systematically evaluate specific psychosocial variables implicated by these theories (e.g., time perspective, perceived control, social comparison, and interpersonal embeddedness) [1, 6, 8, 9, 39, 47] to examine their relative contributions to the observed age patterns. In the long run, a better understanding of these variables could be leveraged to promote continued HRQoL even among those individuals who face considerable functional health threats in their later years.

## Data Availability

The datasets generated and/or analyzed during the current study are not publicly available due to participant confidentiality.

## Abbreviations

ADL: Activities of daily living
CES-D: Center for Epidemiologic Studies Depression Scale
ELSA: English Longitudinal Study of Aging
HALST: Healthy Aging Longitudinal Study in Taiwan
HRQoL: Health-related quality of life
LOESS: Locally regression smoothing method
MCS: Mental component summary score
MMSE: The Mini-Mental State Examination
PCS: Physical component summary score
SES: Socioeconomic status
SF-12v2: 12-item Short Form Health Survey (version 2)
SF-36: 36-item Short Form Health Survey
SPPB: Short Physical Performance Battery
